# Effect of Intranasally Delivered rh-VEGF165 on Angiogenesis Following Cerebral Hypoxia-Ischemia in the Cerebral Cortex of Newborn Piglets

**DOI:** 10.3390/ijms18112356

**Published:** 2017-11-07

**Authors:** Amit Jain, Panagiotis Kratimenos, Ioannis Koutroulis, Amishi Jain, Amulya Buddhavarapu, Jahan Ara

**Affiliations:** 1 Department of Pediatrics, Drexel University College of Medicine, St. Christopher’s Hospital for Children, Philadelphia, PA 19134, USA; amit.jainms@gmail.com (A.J.); ikoutrouli@childrensnational.org (I.K.); amulya.buddhavarapu@gmail.com (A.B.); jahanara224@gmail.com (J.A.); 2 Department of Pediatrics, Sanford School of Medicine, University of South Dakota, Sanford Children’s Hospital, Sioux Falls, SD 57105, USA; 3 Department of Pediatrics, Division of Neonatology, Children’s National Medical Center, School of Medicine and Health Sciences, George Washington University, Washington, DC 20010, USA; 4 Department of Pediatrics and Emergency Medicine, Children’s National Medical Center, School of Medicine and Health Sciences, George Washington University, Washington, DC 20010, USA; 5 College of Pharmacy and Allied Health Professions, South Dakota State University, Brookings, SD 57007, USA; amishijain@gmail.com; 6 Department of Pediatrics, Driscoll Children’s Hospital, Texas A&M College of Medicine, Corpus Christi, TX 77807, USA

**Keywords:** VEGF, hypoxia-ischemia, angiogenesis, HIE, neonate, neurology, hypoxia

## Abstract

Background: Vascular endothelial growth factor (VEGF) stimulates vascular genesis and angiogenesis. Cerebral Hypoxia-Ischemia (HI) leads to the reduction of vasculature in the cerebral cortex of newborn piglets. Objective: The present study tests the hypothesis that post-hypoxia intranasal administration of recombinant human VEGF_165_ (rh-VEGF165) for 3 days increases the vascular density in the cerebral cortex of newborn piglets without promoting neovascularization. Design/Methods: Ventilated newborn piglets were divided into three groups (*n* = 5/group): normoxic (Nx), hypoxic-ischemic (HI), and HI treated with intranasal rh-VEGF165rh-VEGF165 (HI-VEGF). HI piglets were exposed to HI (0.05 FiO2) for 30 min. Recombinant h-VEGF165 (100 ng/kg) was administered 15 min after HI and then once daily for 3 days. The animals were perfused transcardially and coronal brains sections were processed for Isolectin, Hoechst, and ki-67 cell proliferation marker staining. To assess the vascular density, 30–35 fields per animal section were manually counted using image J software. Results: The vascular density (vessels/mm^2^) was 42.0 ± 8.0 in the Nx group, 26.4 ± 4.8 (*p* < 0.05 vs. Nx) in the HI group, and 46.0 ± 11.9 (*p* < 0.05 vs. HI) in the HI-VEGF group. When stained for newly formed vessels, via Ki-67 staining, the vascular density was 5.4 ± 3.6 in the Nx group (*p* < 0.05 vs. HI), 10.2 ± 2.1 in the HI group, and 10.9 ± 2.9 in the HI-VEGF group (*p* = 0.72 vs. HI). HI resulted in a decrease in vascular density. Intranasal rh-VEGF165rh-VEGF165 resulted in the attenuation of the HI-induced decrease in vascular density. However, rh-VEGF165 did not result in the formation of new vascularity, as evident by ki-67 staining. Conclusions: Intranasal rh-VEGF165 may prevent the HI-induced decrease in the vascular density of the brain and could serve as a promising adjuvant therapy for hypoxic-ischemic encephalopathy (HIE).

## 1. Introduction

Hypoxic–ischemic (HI) injury to the prenatal and perinatal brain is a major contributor to global child mortality and morbidity [1,2,3,4]. Perinatal hypoxic–ischemic injury affects between 1 and 8 per 1000 full-term infants and nearly 60% of low birth-weight premature infants [2]. Birth asphyxia is the cause of 20% to 50% of all neonatal deaths worldwide. Approximately 25% of children who survive birth asphyxia develop permanent neurological dysfunctions including cerebral palsy, mental retardation, learning disabilities, and epilepsy [3]. Although the exact cause of neonatal encephalopathy is not always identified, antecedents include prolapsed umbilical cord, uterine rupture, placental damage, maternal hypotension, and acute neonatal and maternal hemorrhage. The outcome from HI injury is further influenced by a variety of factors that include the gestational age as well as the nature, severity, and duration of hypoxic–ischemic insult [5]. Despite advances in supportive care, no effective therapeutic strategies for HI brain injury are available at present and only limited attenuation of injury may be possible using hypothermia in term neonates with moderate hypoxic–ischemic injury [3,4,5,6]. 

Among other molecules that have been used experimentally, mainly targeting the regulatory points of the apoptotic cascade in the area of focal adhesions [7,8,9], Vascular Endothelial Growth Factor (VEGF) has been considered as a potential neuroprotective therapy for neonatal HI brain injury. VEGF is a well-known endothelial cell mitogen as well as a vascular growth and permeability factor with therapeutic potential in ischemic disorders, including stroke [10,11].

VEGF binds to two high-affinity receptors, fms-like tyrosine kinase (flt-1) and the kinase domain region (KDR). Administration of recombinant human VEGF_165_ (rh-VEGF165) improves myocardial perfusion in patients with coronary ischemia and enhances angiogenesis in animal models of myocardial and limb ischemia. Middle cerebral artery (MCA) occlusion in rats evokes expression of VEGF in the ischemic brain, suggesting that after a stroke VEGF may be involved in angiogenesis [12]. Since prolonged hypoxia-ischemia is the underlying cause of both stroke and HI neonatal encephalopathy, we expect similar observations to occur following HI brain injury in neonates. In the ischemic rat brain, when Sun et al. administered exogenous VEGF, they noticed that there was an early neuroprotective effect that reduced infarct size, promoted the survival of nascent neurons, and that the effect became more apparent at 3 and 28 days. The authors reported that exogenous VEGF stimulated angiogenesis in the ischemic penumbra, but not in neuroproliferative zones remote from the site of ischemia. The concern remained that although direct neuroprotection may reduce ischemic injury in the acute phase, neurogenesis, angiogenesis, or both may contribute to longer-term repair of the injured brain [13]. Using magnetic resonance imaging (MRI), three-dimensional laser-scanning confocal microscopy, and functional neurological tests, Zhang et al. measured the effects of administrating recombinant human VEGF165 (rh-VEGF165) on angiogenesis, functional neurological outcome, and Blood Brain Barrier (BBB) leakage in a rat model of focal cerebral embolic ischemia. The authors noticed that late administration of rh-VEGF165 to the ischemic rats (at 48 h post injury) enhanced angiogenesis in the ischemic penumbra and significantly improved neurological recovery. However, early post ischemic (1 h) administration of rh-VEGF165 to ischemic rats significantly increased BBB leakage, hemorrhagic transformation, and ischemic lesions [14]. The effect of exogenous rh-VEGF165 on cerebral cortex post hypoxic ischemic condition in the newborn period has not yet been tested. Due to the acute nature of the perinatal events in newborns and since the occurrence of HI brain injury cannot be predicted beforehand, a non-invasive therapy would be ideal [15,16].

Although VEGF has potent and diverse effects on endothelial cells and neurons, there are significant concerning effects of this agent, such us its actions on vasculature causing hyper-permeable capillaries and larger vessels with questionable functionality, changes on the ependymal cells and hydrocephalus in rodents, and off-target neuroendocrine changes [14,17,18]. So far, the effects of VEGF concentration and the time point for VEGF administration on neuroprotection and angiogenesis in the newborn brain after hypoxic-ischemic injury are not well defined [19,20,21,22]. 

In an attempt to minimize the possible systemic toxicity of the rh-VEGF165 and achieve rapid administration at the time of birth, the current study aims to investigate the effect of intranasally administered rh-VEGF165 on angiogenesis in the cerebral cortex of newborn piglets following cerebral hypoxia-ischemia.

## 2. Results

**Figure 1:**
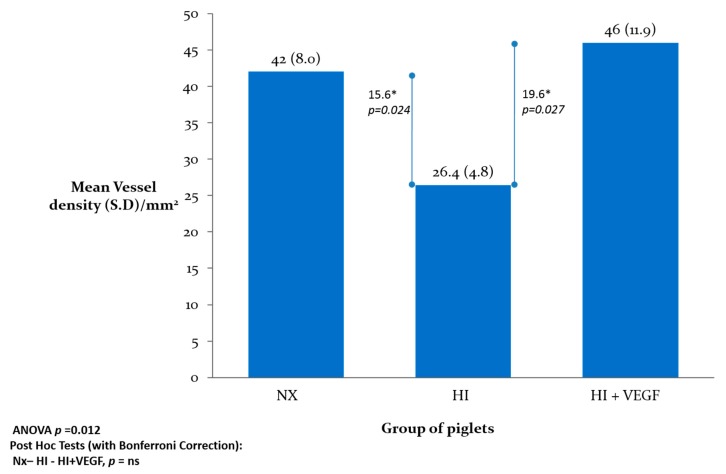
Graphical representation of vascular density in the groups of piglets expressed in vessels/mm^2^. Hypoxic-Ischemic (HI) injury resulted in a decrease in vascular density in newborn piglet brain and treatment with rh-VEGF165 preserved the vascular density.

**Figure 2:**
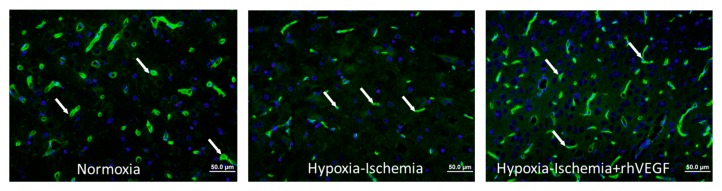
Representative immunohistochemistry staining with (from left to right) Hoechst Nuclear stain, (the arrow demonstrates nucleus), isolectin endothelial cell marker, arrow pointing to blood vessel), and merged images showing Isolectin-FITC (green) around the nucleus (blue) indicating a blood vessel (arrow).

The vascular density (vessels/mm^2^) was 42.0 ± 8.0 in the Nx group, 26.4 ± 4.8 (*p* < 0.05 vs. Nx) in the HI group, and 46.0 ± 11.9 (*p* < 0.05 vs. HI) in the HI-VEGF group (Figure 1). Hypoxic ischemic injury resulted in a significant decrease in vascular density. However, intranasal administration of rh-VEGF165 resulted in a significant increase in vascular density post hypoxia-ischemia. When stained for newly formed vessels, using Ki-67 proliferation marker staining, the vascular density was 5.4 ± 3.6 in the Nx (*p* < 0.05 vs. HI), 10.2 ± 2.1 in the HI, and 10.9 ± 2.9 in the HI-VEGF groups (*p* = 0.72 vs. HI) (Figure 2). Treatment with rh-VEGF165 was not associated with the formation of new vascularity, as evident by ki-67 staining.

## 3. Discussion

Hypoxic insult of the term and preterm neonatal brain is associated with the occurrence of cerebral edema due to vascular leakage and increased expression of vascular permeability markers such as the vascular endothelial growth factor (VEGF) [23,24]. In our laboratory, we used the newborn piglet model of hypoxic encephalopathy that shares multiple morphological and functional characteristics with the human neonatal brain. The distribution of brain injury following global hypoxia in piglets is similar to the distribution of injury in the human brain [25]. Newborn piglets offer a large brain size, the presence of gyri and sulci, similar white/grey matter ratio, and developmental age at term similar to the human brain. Our laboratory [26] and other investigators [25,27,28,29,30,31,32,33,34,35,36,37] have used the newborn piglet model as it provides the advantage of real-time measurements of physiologic parameters such as arterial blood gases and continuous blood pressure monitoring, which allows titration of FiO_2_ to achieve a precise and reproducible degree of hypoxia-ischemia [25,27,32,38]. 

VEGF is a potent factor that increases microvascular permeability to blood plasma proteins within minutes after exposure [39]. The VEGF gene expression is upregulated by hypoxia [40,41]. Numerous studies have highlighted neurotrophic abilities of VEGF, through axonal outgrowth and cell survival following hypoxia in vitro. Persistent or transient hypoxia seems to be associated with increased expression of VEGF in the brain. Topical application of VEGF reduces brain infarct size, and systemically administered VEGF improves neurological outcome from ischemia in rats [40].

In a mouse stoke model, there was a significant increase in the levels of VEGF mRNA and protein in the mouse brain that correlated with the severity of the hypoxic stimulus [42]. Inhibition of VEGF activity by a neutralizing antibody completely blocked the hypoxia-induced increase in vascular permeability, indicating that VEGF is responsible for the hypoxia-induced augmentation in vascular leakage following tissue hypoxia [20,43].

Whether administration of rh-VEGF165 to the ischemic brain has the potential to promote angiogenesis and thereby improve functional neurological outcome has not been adequately tested. Administration of rh-VEGF165 after focal cerebral ischemia may exacerbate blood-brain barrier (BBB) permeability [14]. Following cerebral ischemia, disruption of the BBB occurs acutely, whereas regeneration of cerebral micro vessels develops relatively late in ischemic brain [20,24,44]. The mode of administration is very important, especially for the treatment of HI injury in newborns, a condition that merits very fast delivery of the molecule within a very narrow window of time in order to prevent non-reversible brain injury [15]. There have been several reports of delivery methods of therapeutic agents into the brain through the nasal cavity. The theories that have been proposed include the intraneuronal and the extraneuronal pathways through which the nasally administered molecules travel either with axonal transport from the nasal nerve endings to the brain through the cribriform plate (intraneuronal), or through the intercellular spaces (extraneuronal), finally reaching the brain by bypassing the blood-brain barrier [45,46]. 

In recent years, VEGF has been demonstrated to have multiple roles in developing and adult nervous systems by acting on blood vessels, glia, and neurons. Because of its multiple effects, VEGF treatment may be beneficial for ischemic disorders by inducing angiogenesis and enhancing neuronal plasticity and survival [40,47]. It is well documented that transient and permanent middle cerebral artery (MCA) occlusion upregulates the expression of VEGF in the ischemic brain [48]. The increased expression of VEGF is believed to induce angiogenesis, neurogenesis, chemotaxis of inflammatory cells, and inhibition of apoptosis. Animal and human studies have shown that endogenous angiogenesis plays an important role in improving brain tissue recovery and functional outcome after ischemic stroke. Scafidi et al., using an established mouse model of very preterm brain injury, demonstrated that selective overexpression of human Epidermal Growth Factor Receptor (EGFR) in oligodendrocyte lineage cells or the administration of intranasal heparin-binding Epidermal Growth Factor (EGF) immediately after injury decreases oligodendroglia death, enhances the generation of new oligodendrocytes from progenitor cells, and promotes functional recovery [49]. Recent reports have established that VEGF also has a significant neuroprotective effect on neurons and glial cells, and stimulates their growth and survival [44,50,51]. Because of VEGF’s potent and diverse effects on endothelial cells [52] and the nervous system, a challenge for current and future research is to clarify the usefulness of this growth factor as a therapeutic agent for ischemic injury and stroke intervention, particularly in regard to its angiogenic and neuroprotective capacities [3,5,23,44,48].

As the occurrence of HI is unpredictable, the use of agents such as rh-VEGF165 may provide an adjunct therapy to hypothermia in the post-HI period. We suggest that targeting the cell death pathway and maintaining adequate vascular density by preventing the HI -induced decrease of brain vasculature with agents such as rh-VEGF165 may provide some degree of neuroprotection without promoting new vessel formation and could serve as a promising therapy for Hypoxic-Ischemic Encephalopathy (HIE).

## 4. Materials and Methods

### 4.1. Experimental Procedures

The experimental animal protocol was approved by the Drexel University Institutional Animal Care and Use Committee (IACUC, No. 17757 (30 September 2007) and No. 17716 (30 September 2008)) and performed in accordance with US National Institutes of Health guidelines as outlined in the Policy on Humane Care and Use of Laboratory Animals (NIH publication, August 2002).

Newborn piglets were divided into three groups (*n* = 5/group): Normoxic (Nx), hypoxic-ischemic (HI), and HI treated with rh-VEGF165 (HI-VEGF). The Nx piglets were maintained in 0.21 FiO_2_ and normal blood pressure, while the HI piglets were subjected to a combination of hypoxia (0.05 FiO_2_) for a pre-defined period of 30 min and ischemia induced by a 10-min period of hypotension. In the HI-VEGF group, the recombinant human VEGF165 (100 ng/kg) was administered 15 min after HI and then once daily for 3 days.

### 4.2. Induction of Cerebral Hypoxia–Ischemia

The hypoxia-ischemia (HI) model used in the study is based on Bjorkman et al. [38] with some modifications, as described in our previous studies by Ara et al., 2011 and 2013 [21,22]. Newborn female piglets (1 day old with an average weight of 1.5 kg) were anesthetized with 4% isoflurane. The piglets were intubated with an endotracheal tube and ventilation was initiated using a mechanical ventilator with initial settings of 20 breaths per min (bpm), peak inspiratory pressure (PIP) 25 cm, positive end expiratory pressure (PEEP) 5 cm, and inspiratory time 0.65 s. Inspired oxygen (FiO_2_) and PIP were adjusted to maintain arterial oxygen saturation (SaO_2_) at 95%–98% and arterial pCO_2_ at 35–45 mm Hg. An umbilical artery was aseptically cannulated with a neonatal umbilical catheter to monitor the blood pressure and arterial blood gasses. Core body temperature was maintained at 38–39 °C with an overhead radiant heater. The temperature was maintained within normal range during the HI insult. Heart rate (HR), mean arterial blood pressure (MAP), temperature, and SaO_2_ were monitored and recorded for the duration of the experiment. A digital electroencephalogram (EEG) device was used to monitor electroencephalography (EEG) amplitude and frequency. After the endotracheal intubation, the use of isoflurane was discontinued, and fentanyl (0.05 mg/kg) and pancuronium (0.3 mg/kg) were given throughout the experiment as needed to maintain anesthesia. The pancuronium eliminated spontaneous breathing and was used in an attempt to achieve the maximum control of the ventilation of the animals, closely following physiologic parameters and arterial gases. The animals were fully anesthetized during the hypoxia and instrumentation as well as following the re-oxygenation and brain harvesting in order to minimize any pain or discomfort. Pressure support setting was used for the ventilation. After a stabilization period of 30 min, the piglets were assigned to either the normoxic (0.21 FiO_2_) or hypoxic–ischemic group. Hypoxia–ischemia was induced by decreasing FiO_2_ to 0.05 and continued for 40 min. FiO_2_ was decreased or increased by 0.01 increments during the insult to maintain HR (>130 beats/min) and MAP (>70% baseline). Upon reinstatement of HR or MAP, FiO_2_ was returned to 0.05. Hypotension was induced for the final 10 min of the insult by decreasing FiO_2_ until the MAP was <70% of baseline. Hypoxia was terminated by resuscitation with 100% oxygen. Metabolic acidosis was half-corrected by administering sodium bicarbonate to maintain extracellular physiologic pH. Following 10 min of 100% O_2_, the ventilator rate and FiO_2_ were gradually reduced to maintain PaO_2_ within the normal range until the piglet was able to breathe spontaneously, at which time the piglet was extubated following close observation. A single dose of vancomycin 10 mg/kg was administered intravenously at the end of the experiment to prevent infection. 

### 4.3. Intranasal Administration of Recombinant Human VEGF_165_

After the induction of hypoxic ischemic injury and post stabilization, the piglets were anesthetized with 4% isoflurane and placed in a supine position. The head was stabilized in a horizontal direction with a soft neck roll. A pipette (P100) was used to intranasally administer 5–10 µL drops of vehicle (0.9% NaCl) or rh-VEGF165 dissolved in 0.9% NaCl to alternating nostrils every min. A total of 200 µL of dosing solution of rh-VEGF165 (100 ng/kg body weight) was delivered over a course of 8–10 min. The rh-VEGF165 was administrated 15 min after HI and then once daily for 3 days. Subsequently, the piglets were perfused transcardially; the brain was harvested and coronal brains sections were processed for histological and immunohistochemical analysis.

### 4.4. Immunohistochemistry

Immunohistochemistry staining was performed as previously described by Ara et al., 2011, 2013 [21,22]. Briefly, coronal brain sections were de-paraffinized in xylene and rehydrated in graded ethanol and distilled water. High temperature antigen retrieval was performed in 10 mM sodium citrate buffer. Sections were blocked and incubated with primary antibodies green fluorescent Alexa Fluor^®^ 488 isolectin GS-IB4 conjugate (an endothelial cell marker, Invitrogen, Carlsbad, CA, USA). The sections were washed in PBS and counterstained with the nuclear dye Hoechst 33258 (2 μg/mL in PBS) for 5 min, and subsequently with cell proliferation marker ki-67. All sections were examined using an Olympus DP-70 digital camera mounted on an Olympus IX70 inverted microscope connected to a computer with Olympus multifunctional software for digital image analysis.

### 4.5. Vascular Density Assessment

**Figure 3:**
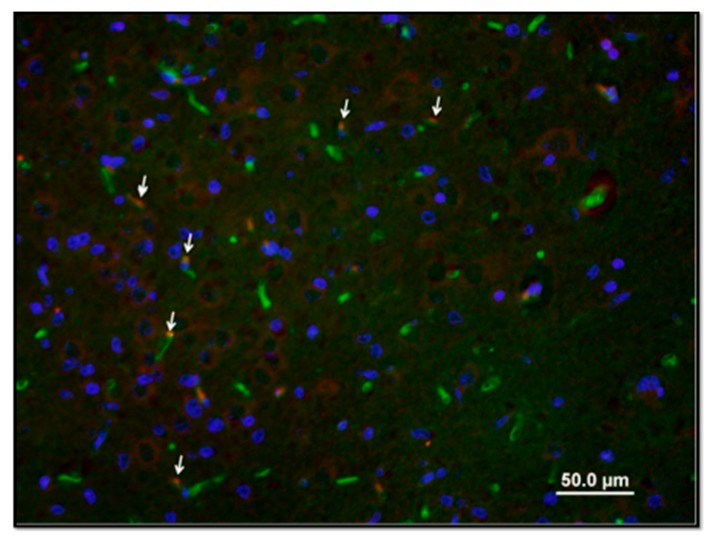
Representative image of immunostaining with ki-67 cell proliferation marker demonstrating the newly formed vessels (red) and merged images showing Isolectin-FITC (green) around the nucleus (blue), indicating a blood vessel. Note the nuclei co-localization with endothelial cells (arrows).

The potential effect of rh-VEGF165 on blood vessel density was assessed by isolectin GS-IB4 (endothelial cell marker) immunostaining (Figure 2). Similar brain sections on the same levels were stained along the three groups and specifically on the cortex, hippocampus, and putamen. In each section, 30–35 fields were captured to analyze the entire slide. Isolectin GS-IB4, nuclear dye Hoechst 33,258 images, and ki-67 cell proliferation marker were superimposed using Olympus image mixture software for the accurate assessment of vascular density (Figure 2 and Figure 3). Vascular density was then determined by the manual counting of vascular structures on each section field by Image J software (Bethesda, MD, USA) and expressed per mm^2^. [53,54] Immunostaining quantification, which was performed by two different investigators blinded to treatment and clinical course, was conducted in two specific areas identified using a pig brain atlas, namely, the CA1 region of the ventral hippocampus and the putamen and cortex [55].

### 4.6. Statistical Analysis

Mean vascular density was determined in each animal and then mean ± standard deviation (M ± SD) was calculated for each of the groups. All values are presented as mean ± standard deviation (SD). The mean immunohistochemistry scores were compared using Kruskal-Wallis One Way ANOVA on ranks followed by the Bonferroni multiple comparisons test. Differences with *P* values of <0.05 were considered as significant.

### 4.7. Study Limitations

We were unable to include a control group of normoxic +rhVEGF animals. We were unable to use in vivo antibodies against VEGF, since they did not cross the blood-brain barrier. Only female piglets were included in the study and there was no randomization based on the gender of the animals.
